# Food waste accounting methodologies: Challenges, opportunities, and further advancements

**DOI:** 10.1016/j.gfs.2019.01.002

**Published:** 2019-03

**Authors:** Sara Corrado, Carla Caldeira, Mattias Eriksson, Ole Jørgen Hanssen, Hans-Eduard Hauser, Freija van Holsteijn, Gang Liu, Karin Östergren, Andrew Parry, Luca Secondi, Åsa Stenmarck, Serenella Sala

**Affiliations:** a European Commission, Joint Research Centre (JRC), Via E. Fermi, 2749, 21027 Ispra, VA, Italy; b Swedish University of Agricultural Science, Department of Energy and Technology, Box 7032, SE-75007 Uppsala, Sweden; c Ostfold Research, Stadion 4, NO-1671 Kråkerøy, Norway; d European Commission, Directorate-General Eurostat, ESTAT.E.2 - Environmental Statistics and Accounts, Sustainable Development; 5, rue Alphonse Weicker, L-2721 Luxemburg, Europe; e VHK BV, Elektronica weg 14, 2628 XG Delft, The Netherlands; f SDU Life Cycle Engineering, Department of Chemical Engineering, Biotechnology, and Environmental Technology, University of Southern Denmark, 5230 Odense, Denmark; g RISE-Research Institutes of Sweden, Agrifood and Bioscience, Ideon Gateway, SE-22370 Lund, Sweden; h Lund University, Department of Food Technology, Engineering and Nutrition, Box 124, SE-22100 Lund, Sweden; i WRAP, Blenheim Court, 19 George Street, Banbury, Oxon OX16 5BH, United Kingdom; j University of Tuscia, Department for Innovation in Biological, Agro-Food and Forest Systems (DIBAF), Via S.Camillo De Lellis, snc, 01100 Viterbo (Vt), Italy; k IVL Swedish Environmental Research Institute, Box 21060, SE-10031 Stockholm, Sweden

**Keywords:** Food waste, Food loss, Accounting methodology, Systemic approach, Sustainable development goal 12.3, Policy support

## Abstract

About one third of the food produced globally is wasted along the food chain, representing a burden for the environment and an inefficiency of the food system. Tackling food waste is a priority on the global political agenda to guarantee food security. Defining a methodology for food waste quantification is key to monitoring progress towards the achievement of reduction targets. This paper summarises the outcomes of a workshop on food waste accounting co-organised by the European Commission's Joint Research Centre and Directorate-General on Health and Food Safety with the aim of stimulating harmonisation of methodologies, identifying challenges, opportunities, and further advancement for food waste accounting. The paper presents methodological aspects, e.g. system boundaries, reliability of data, accounting of water flows, to ensure better support to food waste policy design and interventions. It addresses all the actors of the food supply chain, governments, and research institutions.

## Highlights

• Methodology for waste accounting is key for monitoring progress towards reduction targets.
• Challenges, opportunities, and further advancements for food waste accounting methodologies are discussed.
• The harmonisation of accounting approaches is one of the main challenge.
• Existing tools are opportunities to strengthen the efficacy of food waste accounting.
• Further advancements include the increased availability of data, especially on liquid waste.

## 1 Introduction

About one third of the food produced globally is lost or wasted along the food supply chain (FSC), from primary production up to consumption (FAO, 2011). This figure represents a considerable inefficient use of valuable resources within the food system, contributing to natural resource depletion and environmental pollution while undermining food security (Kummu et al., 2012). The economic impact of food waste (FW) is substantial as well (FAO, 2013). Reducing food loss along the supply chain and halving FW at the consumption and retail stages by 2030 is one of the targets of the Sustainable Development Goals (SDGs) envisaged by the 2030 Agenda for Sustainable Development adopted in 2015 namely the SDG 12.3 (UN, 2015). At European scale, the commitment to reduce FW generation has been declared in the European Circular Economy Action Plan (EC, 2015), and in the directive amending the European Waste Framework Directive (European Parliament and Council, 2018), in line with the SDG 12.3 target.

FW accounting is a central element for FW policy design and interventions. It shapes the baseline against which the achievement of reduction targets should be evaluated, allows for the monitoring of FW generation over time, and, together with other tools, supports the prioritisation of actions through the identification of the most relevant waste streams. Nonetheless, FW accounting is a complex task. Past experiences on FW accounting highlighted that existing data are characterised by significant uncertainty, due e.g. to the limited representativeness of the sample in which primary data are collected. In addition, the different methodological approaches adopted address different types of “loss” or “waste”, leading to results which are not comparable (Corrado and Sala, 2018, Xue et al., 2017). These features may limit the usefulness of FW accounting results as a basis for monitoring reduction progress over time and informing decision-making processes. Indeed, the absence of a consolidated methodological approach could undermine a deep understanding of the results from the recipient of the information, who, e.g., can misinterpret the streams of FW included in the accounting process.

In this paper, the definition of FW reported in the FUSIONS definitional framework (Östergren et al., 2014) is adopted, which states that “food waste is any food, and inedible parts of food, removed from the food supply chain to be recovered or disposed”.

Several ongoing initiatives are helping to fill the gap between the need for a robust estimation of FW generation and the lack of a harmonised approach to account for it. In 2016, a multi-stakeholder initiative (Food Loss and Waste (FLW) Protocol) was launched with the aim to develop an internationally accepted FW accounting and reporting standard. Part of the efforts of this initiative were formalised in the “Food Loss and Waste Accounting and Reporting Standard” (FLW Standard) published in 2016 (Food Loss and Waste Protocol FLW Protocol, 2016), which provides guidance on FW accounting for governments, companies, and other entities. In the same year, a coalition of executives from governments, businesses, international organisations, research institutions, farmer groups, and civil society was formed under the name 'Champions 12.3', with the objectives to inspire ambition, mobilise action, and accelerate progress towards the achievement of the SDG 12.3 on FW reduction (World Research Institute WRI and Ministry of Economic Affairs of the Netherlands, 2016). In September 2017, Champions 12.3 released guidance on the interpretation of SDG 12.3, to avoid ambiguity about definitions, life cycle stages, types of materials, destinations, and monitoring indicators to be considered for the achievement of the 12.3 reduction target (Champions 12.3, 2017).

A joint effort to harmonise FW quantification approaches is also ongoing at the European scale. The FUSIONS (Food Use for Social Innovation by Optimising Waste Prevention Strategies) project, financed under the European Union's Seventh Framework Programme for research, technological development and demonstration, has delivered an extensive overview of available methodologies at different stages of the food chain (Møller et al., 2014a, Møller et al., 2014b), a definitional framework for FW (Östergren et al., 2014), and a quantification manual (Tostivint et al., 2016). The FUSIONS manual, coherent with the principles of the FLW Standard, should be applied at the national scale with the objective of guiding European Member States in the quantification of FW. In addition, the European Commission established in 2016 the EU Platform on Food Losses and Food Waste. It includes a working group on FW measurement, bringing together experts from relevant organisations from Member States, with the aim to report against the requirements of the European Circular Economy Action Plan (EC, 2015), and monitor progress towards SDG 12.3.

With the objective of strengthening the harmonisation of FW accounting methodologies applied by different actors of the supply chain, the European Commission's Joint Research Centre (JRC) and Directorate-General for Health and Food Safety (DG SANTE) co-organised the workshop “FW accounting: methodologies, challenges and opportunities”, held on 26th of September 2017 in Brussels. The focus of the workshop was on challenges related to the improvement of FW accounting in support to the decision-making processes, existing opportunities to overcome these challenges, and further advancements, both from practical and research sides, which have the potential to improve FW quantification at different geographical scales.

This paper summarises the main outcomes of this workshop, integrating them by evidence and critical considerations reported in literature. It presents an overview of challenges, opportunities and further advancements in the context of FW accounting. The paper has the aim of stimulating the harmonisation of methodologies, capitalising on existing experiences, towards ensuring more efficient decision support to FW policy design and interventions.

## 2 Context of this study

The workshop “FW accounting: methodologies, challenges and opportunities” brought together 34 experts on FW, from public and private organisations (Caldeira et al., 2017). Specifically, 40% of participants were from research centres, 24% from academia, 24% from other public institutions, and 12% from the private sector. The discussion at the workshop covered two main topics: the challenges that FW accounting has to overcome to effectively support the decision making process towards FW reduction, and the lessons learnt from experiences on FW accounting at European, national, or company scale, considering different types of accounting approaches.

The contents of this paper reflect the topics discussed in the workshop complemented by relevant literature. All the authors, who took part at the workshop, have agreed on the subjects of this paper.

## 3 Overview of the main challenges, opportunities and further advancements

Starting from the presentation of previous experiences, thirteen challenges were identified for FW accounting. Moreover, opportunities and further advancements were discussed for each of them, as summarised in Table 1 and reported in the next sections. It is worth noticing that the challenges are meant to represent the objectives of FW accounting, the opportunities are the tools which are currently available to meet them, and further advancements refer to the means, which may support future better accounting.

**Table 1:** Summary of challenges, opportunities, and further advancements to be addressed in food waste (FW) accounting.

**Food waste accounting elements**	**Challenges**	**Opportunities**	**Further advancements**
Study aim and definitions	– Have a clear aim of the study to select the most suitable quantification method	– Rely on existing tools or documents for the selection of the most suitable method, e.g. "FLW quantification ranking tool", FLW Standard, FUSIONS quantification manual, "Food waste measurement principles and resources guide"	
	– Use FW-related terminology and definitions in an harmonized way	– Refer to existing guidelines with definitions and indications for system boundaries – Use of examples on what is considered edible and inedible	– Develop internationally agreed definitions consistently applied
	– Have a clear understanding of the amount of by-products sent to valorisation routes, e.g. animal feed and biomaterials		– Increase data availability, including engaging feed industries or by-products suppliers to feed system
	– Have an harmonized approach in the definition of system boundaries to account for FW generation	– Rely on recommendations on system boundaries in existing reports/guidelines, e.g. FLW Standard, FUSIONS quantification manual	– Improve data availability on dynamics of generation of waste at the primary production stage
Data collection	– Ensure representativeness of primary data, which may be affected by: high variability (e.g. high number of small entities), underestimation of FW, omission of liquid waste and home composting, difficulties in having data from industries	– Rely on comparison of quantification methods to collect primary data on consumer waste that showed that self-reporting, kitchen caddies, and coding of photographs have the best potential – Build on existing best practices at the national level	– Increase primary data availability – Develop guidelines to select representative samples for data collection and cover seasonal variability
	– Use secondary data taking into account their degree of representativeness	– Introduce proper statistical methods and estimators able to deal with local-level estimations	– Combine primary and secondary data to get a more detailed picture
	– Have a robust knowledge of FW generation both from a territorial and consumption –based perspectives	– Consider territorial and consumption-based approaches which may serve complementary purposes, e.g. assessing the overall amount of FW associated with a consumption pathway	
Water flows accounting	– Establish a framework to account for moisture content variation in food products along the food supply chain and over time	– Use alternative units of measurement, e.g. dry mass, energy content – Rely on recommendations on which type of water flows to be included in the accounting in existing reports/guidelines, e.g. FUSIONS quantification manual	– Increase availability of data on variation of moisture content of different products along the food supply chain – Account separately for FW (with embedded water) and water lost (evaporated) or added along the food supply chain – Develop models for estimating water content in FW streams
	– Have a robust knowledge of the liquid FW generated during food manufacturing and sent to sewers in food services/households		– Increase the availability of data on liquid waste, considering differences between industries, which may have an internal wastewater treatment facility, and households
Uncertainty	– Reduce as far as possible and deal with statistical uncertainties	– Report uncertainty ranges of estimation – Rely on material flow analysis and double checks with other sources of data (e.g. food left for consumption)	– Increase data availability to reduce statistical uncertainty
	– Reduce as far as possible statistical uncertainties and clearly report them	– Rely on existing methodological guidelines to limit methodological uncertainties	– Develop sector-specific guidelines that are coherent with general ones (e.g. FLW Standard and FUSIONS quantification manual) – Define a framework to assess uncertainties
Policy needs	– Identify the best indicators to meet specific policy needs	– Use different types of indicators already applied in other studies, e.g. economic value and dry mass may provide different but complementary information	
FW generation drivers	– Have a solid understanding of the relationship between drivers and FW generation, encompassing behavioural, socio-economic, cultural, contextual aspects		– Increase knowledge on FW generation drivers, including coupling socio-economic and behavioural science-based assessment

### 3.1 Study aim and definitions

The aim of the accounting exercise, e.g. to increase FW prevention, has a crucial role in shaping the type of features of FW, e.g. edibility, that should be grasped. The specific context significantly influences the choice of the methodology: quantifying FW in a company to increase the efficiency of the production process, for example, is different from quantifying FW in a region to support food security, and the two tasks face different challenges. Overall, clearly defining the aim of the study is the first step towards an effective design and a proper choice of the quantification method. For this purpose, the FLW Protocol has developed the “FLW quantification ranking tool”, which helps users identify the method best suited to them (Food Loss and Waste Protocol FLW Protocol, 2018). Furthermore, other documents, such as the FLW Standard (Food Loss and Waste Protocol FLW Protocol, 2016), the FUSIONS quantification manual (Tostivint et al., 2016), the “Food waste measurement principles and resources guide” (WRAP, 2018a, WRAP, 2018b), and the sector-specific guidance published by WRAP for the hospitality sector (WRAP, 2018b) provide comparative evaluation of methodologies and suggestions to help steer the choice.

Other key elements of the accounting are definitions and terminology, encompassing type of FW, system boundaries (e.g. the point of the life cycle from which unconsumed food can be considered FW), and FW destination (Chaboud and Daviron, 2017).

Differences in both the terms used and in the meanings attributed to these terms may limit the comparability of the results from existing studies. For example, a distinction between food loss (defined as occurring before retail) and FW (occurring during retail and consumption) was reported only in some studies (e.g. FAO, 2011; Porter et al., 2016). Furthermore, the terms FW or food loss were defined either on the intended destination of food (FAO, 2011) (e.g. including all the food not consumed by humans), or considering its actual destination (Östergren et al., 2014, Stenmarck et al., 2016) (e.g. food sent to waste treatment facilities). In official European waste statistics accounting, by-products sent to animal feed and biorefineries are also not accounted for. Statistics from the Norwegian ForMat project, for example, indicates that, by using this definition, 70% of all food lost or wasted from the retail sector might not be registered as FW because it is used as animal feed (Stensgård and Hanssen, 2016). The adoption of internationally agreed definitions would strengthen the comparability of studies. Furthermore, although not falling under the definition of FW, a parallel estimation of by-products used for feed and biomaterials would be useful to monitor the trends towards the cascade use of FW. This exercise would be a challenging task due to the lack of data. Companies and by-products suppliers could make a valuable contribution by getting involved.

Wasted food can be classified as edible or inedible and as avoidable or unavoidable. The definition of what is edible/inedible and avoidable/unavoidable may be related both to physical and cultural elements (Redlingshöfer et al., 2017), and depends on the context. The definitional framework of FUSIONS (Östergren et al., 2014) distinguished between edible and inedible FW. According to the FUSIONS manual (Tostivint et al., 2016), only the total amount of FW should be reported, although it could also help to make the distinction between edible and inedible FW. To strengthen the support which FW may provide to the decision-making processes, a clear definition of edible and inedible parts of food should be provided, complemented with examples which may help understand this definition.

System boundaries should be set in the definition of FW. In some cases, FW is considered as the amount of harvested crop lost in the supply chain or during harvesting (FAO, 2011), whereas in others, mature food that is not harvested is also accounted as FW or as other types of flows, e.g. “side flow” (Hartikainen et al., 2017). These differences may affect FW monitoring and the comparison of results from different studies. Specific recommendations on the streams to include in the accounting process are provided by some of the available reports or guidelines (e.g. Östergren et al., 2014). However, further insights are needed, especially for agricultural production, where there are only a few relevant studies (e.g. Redlingshöfer et al., 2017) and for which very few data are available.

### 3.2 Data collection

#### 3.2.1 Primary data

Direct measurements of FW generation along the FSC can include, for example, waste compositional analyses (WCAs), waste register at retail levels, FW diaries at household levels, and questionnaires. Direct measurements can provide a considerable granularity of data and, in some cases, support the analysis of the drivers behind FW generation. However, carrying out such measurements is quite costly, while approaches based on self-reporting can lead to biased results (Parry, 2017). In addition, in case the accounting exercise interests a broad geographical area and FW statistical data cannot be collected in all business units along the food chain nor in all households, it is important to develop clear guidelines on how to select a representative sample of businesses and households to up-scale to national statistics.

WCAs entail complex logistics and capture only the amount of food sent to waste management, excluding, for example, the amount of food disposed of via the drain or home-composted. Furthermore, food is often disposed together with other materials, e.g. packaging, and the quantification of FW may be not straightforward.

Past experiences of FW accounting carried out in the UK highlighted that FW diaries are characterised by an uncertainty level ranging between 12% and 20%. Furthermore, their compilation may lead to underreporting by up to 40% and can be influenced by the characteristics of the individuals reporting the data (Parry, 2017). In a study carried out on a representative sample of European Union citizens and based on self-administered questionnaires, it emerged that individuals with a low level of education were most likely to generate a lower percentage of FW. This finding may be related to this group of people actually generating less FW or to a greater degree of under-reporting, e.g. due to lower levels of engagement in the research process or poorer understanding of the instructions (Secondi et al., 2015). In the REFRESH (Resource Efficient Food and dRink for the Entire Supply cHain) project, different methods for accounting for consumer FW were assessed, and self-reporting, kitchen caddies, and coding of photographs were found to have the best potential (Van Herpen et al., 2016)

Furthermore, most of the life cycle stages are characterised by a large number of small entities with different features, which makes it quite difficult to collect representative data. Within a Swedish study, for example, important differences were found even in FW generated by kitchens belonging to the same organisation (Eriksson et al., 2017a).

The collection of primary data from industry may also present challenges. First, businesses are not always keen on sharing data on FW levels, as this may be linked to a perception of their efficiency and therefore be commercially sensitive. Furthermore, terminology generally used in industries may not coincide with the definitions used in studies on FW and in the legislative framework. Surplus food used for animal feed and by-products, for example, is often classed and reported as waste in the industrial context, whereas it may not in fact be covered by the scope of the relevant definition of FW. Finally, part of the FW may be transformed by industries, e.g. dried, diluted or digested, changing the mass of FW originally generated (WRAP, 2016).

In addition, FW generation along the FSC may also be influenced by seasonal variability due, for example, to the production and consumption of different types of food, and therefore collecting data only at one time of year may lead to an unrepresentative picture of FW generation. An example of this is illustrated in Eriksson (2015) where the aggregated mean weekly variation of FW from six Swedish supermarkets over a period of five years ranged from 0.8% to 4%. Since this is the aggregated waste, the variation over time for individual stores, departments and products is much larger. The Norwegian ForMat project represents a successful example of primary data collection along the FSC. It developed a systematic methodological approach with annual collection of primary data from a number of food industries, covering 25% of the sector based on economic turnover, 50% of the wholesale sector, and a representative number of retail shops and municipalities (Stensgård and Hanssen, 2016). Annual collection of data has proven to be important to establish a common methodological platform, knowledge about the methodologies to be used as well as internal and external interest in the data and statistics. Having a third party organisation collect and manage data, and calculate and report statistics, was important to develop voluntary reporting of data by all actors in the food chain. This study showed that, once the system was put in place, annual updating of statistics for the whole FSC was not very resource intensive. The development of guidelines on how to select a representative sample for data collection and capture seasonal and geographical variability in consumption is considered a possible further advancement for FW accounting.

#### 3.2.2 Secondary data

Data not collected directly from entities generating FW but retrieved from the literature, statistics or other sources, are defined as secondary data. The collection of secondary data is less expensive than the collection of primary data. However, secondary data may not fully match the investigated context, and the users of secondary data generally do not have the ability to control their representativeness. Indeed, the sources of secondary data are often recurring in various studies, and may not be up to date or representative enough for the purpose of the study (Xue et al., 2017).

Therefore, to define a robust and comprehensive picture of FW generation, on the one hand more primary data are needed that can capture (in terms of representativeness) the variability characterising FW generation along the supply chain throughout the year. On the other, the costs of collecting primary data could be prohibitively high, especially for sub-national representative information, i.e. measures related to the individuals’ local area of residence, which represents the local level at which policymakers should act (Secondi et al., 2015). As a result, a compromise between data reliability, representatives and economic commitment should be found. In this regard, data availability for small domains could be improved by the introduction of proper statistical methods and estimators that can deal with local-level estimates.

#### 3.2.3 Territorial and consumption-based approaches for food waste accounting

FW is generally estimated using a territorial-based approach (e.g. FAO, 2011, Stenmarck et al., 2016). In a territorial-based approach, waste generation is allocated to the geographical area where the FW is produced, whereas in a consumption-based approach FW generated along the FSC of food is allocated to the area where the food is consumed (e.g., Merciai and Schmidt, 2018).

The territorial-based approach is relevant when the aim of the accounting exercise is to estimate the actual amount of FW to be managed or valorised in a certain area. When comparing FW generation on this basis between countries, it may disadvantage countries that are large producers and exporters of food products, e.g. seafood in Norway, meat in Denmark and the UK, cheese and other dairy products in the Netherlands, fruit and vegetables in Spain, etc. This is especially relevant if FW is measured and compared on a per capita basis, and different types of indicators need to be used to make “fair comparisons” between countries, e.g. weight of FW per tonne of food produced. The consumption-based approach, instead, represents a valuable approach for considering the FW “embedded” in food products along the FSC, e.g. while assessing FW generation associated with a specific consumption pathway.

### 3.3 Water flows accounting

The amount of water embedded in food products may change considerably along the FSC, e.g. due to temperature variations and cooking practices. At the consumption stage, for example, the mass of some products such as tea bags and rice, can be doubled or trebled, or reduced by the same extent (Yadav and Jindal, 2007). However, water losses occur all along the FSC, particularly in hotter seasons and geographical areas. Direct measurement of FW, therefore, can be influenced by moisture content variations and, existing studies barely report how changes in water content variations were accounted. Indeed, Corrado and Sala (2018) highlighted that even small changes in moisture content variations may considerably influence the overall wet mass of FW.

The FUSIONS quantification manual (Tostivint et al., 2016) provides specific guidance on how to account for water flows. In compliance with the European food law (EP, 2002), water intentionally incorporated into food during its manufacture, preparation or treatment (e.g. water added to fruit juice or water incorporated into rice during cooking) is considered food, and therefore is to be accounted as part of FW when the food itself is discarded. On the contrary, water not incorporated into a product should not be counted as FW, e.g. water used to flush food down the drain during cleaning processes. Water intentionally removed during the processing or preparation of food, e.g. water that evaporates during cooking, baking or drying, should not be considered as FW but unintentionally removed water. The FLW Standard recommends the user to report any changes in intrinsic water content.

An ideal approach to distinguishing between inefficiencies of the food system and mass changes due to moisture variations would be to account separately for the actual mass of unconsumed food products, and for the mass of water lost or added along the supply chain. This may imply a considerable level of uncertainty due to difficulties in accounting for water flows along the FSC. The extent of moisture content variations within life cycle stages needs to be further investigated, and the definition of “water loss/gain” coefficients or models that predict water content in FW streams for specific products and stages of the FSC may support the definition of a comprehensive mass balance. Since variable water content is mainly a methodological challenge for quantification in terms of mass, other units such as energy content, or monetary value (except when products are sold by mass) that are less sensitive to water content could be used. However, these units bring other methodological challenges, e.g. lack of transparency on the amount of FW to be managed and fluctuation over time (for economic value), so there are no perfect units to be used in FW quantification (Eriksson, 2015).

The majority of existing guidelines for FW accounting include liquid food disposed through the sewer (e.g. Tostivint et al., 2016). Data on the amount of food disposed via the sewer are very limited and the need for such kind of primary information is even more critical than for solid FW. Concerning the consumption stage, waste statistics do not grasp this amount of discarded material and errors in self-reporting may be relevant. At the industrial level, part of the food is lost through sludge, which can be highly diluted. Given that industries often do not categorise sludge as FW, it is very complex to reach a robust estimation of this type of flow. Cooperation with industries may help to overcome this challenge.

### 3.4 Uncertainty

FW quantification exercises are characterised by at least two types of uncertainties: statistical, i.e. due to limited representativeness of samples for data collection, and methodological, i.e. associated with methodological assumptions.

In light of the complexity of the food system and of the underpinning dynamics of FW generation, a considerable reduction of statistical uncertainties at the European level would be hugely challenging and highly expensive. It is therefore important to complement estimates as much as possible with information on uncertainty, such as confidence intervals, and refer to ranges of FW generation, rather than to average values. An important measure to be adopted during data collection should be to develop homogenous samples of FW-generating units, to reduce the variation within each group, e.g. different types of food producers. Moreover, the adoption of a systemic approach, based on closed mass balance taking into consideration the whole food system, such as material flow analysis (MFA) (Hendriks et al., 2000), could help refine the overall picture of FW generation. Indeed, it increases the accounting reliability, making sure that all input and output flows are considered, limiting inconsistencies due to uncertainties. A first attempt to obtain a complete picture of EU food flows using MFA was executed by Kemna et al. (2016). The resulting mass flow diagram indicated the amount of food and FW in the different stages of the EU supply chain, with a ± 20% reliability range. Double-checking against other sources of data, such as surveys on food consumption, could help verify reliability of data.

Reducing methodological uncertainties through a shared and clear accounting approach and transparent reporting of conditions and assumptions behind the survey would be very useful for monitoring FW generation over time. Various guidelines have been developed, covering a broad range of applications: general principles (e.g. Food Loss and Waste Protocol FLW Protocol, 2016) and sector-specific (e.g. WRAP, 2018b). It is recommended that existing general guidelines are followed in the development of other sector-specific ones to ensure methodological consistency and streamline the reduction of methodological uncertainties.

Focusing more on the reduction of methodological rather than on statistical uncertainties would be more advantageous. Nevertheless, targets for FW reduction have been defined at the political level (European Commission (EC), 2015, United Nations (UN), 2015) and initiatives at both the global and European levels are underway to define indicators for monitoring FW generation (European Commission (EC), 2018, United Nations Statistics Division (UNSD), 2017). Therefore, a reliable baseline to check progress against targets is needed. Furthermore, a comprehensive and detailed estimation of FW generation would support the prioritisation of actions to tackle FW.

**Fig. 1:**
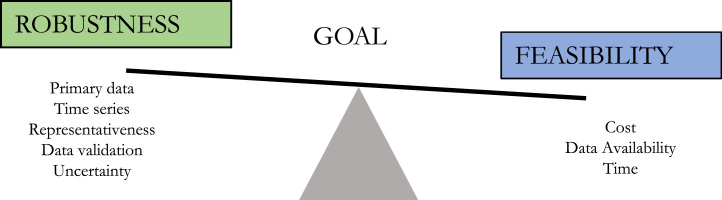
Elements to be considered to balance robustness and feasibility. Obtained from Caldeira et al. (2017).

In light of the specific needs and available resources for the accounting, a balance should be found between the feasibility of the accounting process, e.g. resources needed to refine the FW estimations, and the robustness of the methodological approach, e.g. ability to monitor FW volumes over time (Fig. 1). The “FLW quantification ranking tool” developed by the FLW Protocol may support in the choice of the most apt method (Food Loss and Waste Protocol FLW Protocol, 2018).

### 3.5 Policy needs

Tackling FW is a priority on the global political agenda. Robust accounting of FW is needed to strengthen the definition of a baseline and to monitor progress over time towards FW-related targets. This section presents the elements of the accounting process that should be considered to meet policy needs.

As already mentioned, the study design plays a central role and should be defined according to the aim of the accounting exercise or the policy questions. For example, the distinction between edible and inedible parts of food may be relevant to strategies on food prevention and food security, whose main aim is to reduce edible FW, although they imply a concomitant reduction in associated inedible parts. On the other hand, knowing the composition of FW may be useful to identify pathways for its valorisation, as either energy (e.g. influence of humidity on the calorific value of the FW) or material (e.g. specific waste flows coming from food manufacturing that could be upcycled) (Cristobal et al., 2018). Furthermore, having a detailed picture of FW generation per product or product groups may support the prioritisation of prevention or valorisation interventions (De Laurentiis et al., 2018). A summary of the links between policy interventions and possible accounting needs is reported in Table 2.

**Table 2:** Example of the link between areas of policy interventions and possible accounting needs.

**Areas of policy intervention**	**Accounting needs**
Waste prevention/Food security (e.g. reduction of inefficiencies in the food system)	– Distinction between edible and inedible parts of food – Detailed breakdown in product groups or processes – Link between the quantities and drivers of FW – Inclusion of all types of treatment of food not eaten by human beings or used directly as ingredients of new food products.
Waste management (optimisation of the treatment of FW in treatment facilities)	– Estimation of total amount to be managed and how it is treated (e.g. by anaerobic digestion, composting, incineration) – Assessment of the moisture content – Knowledge of the composition and origin of waste stream
Waste valorisation as energy/material (e.g. using FW to produce biomaterials or biofuels)	– Awareness of the composition of waste streams – Analysis of the extent to which the provision may be steady over time (e.g. seasonality) – Consideration of the origin of FW

At the European level, the statistical system reports waste on a three digit level following the European Waste Classification for statistical purposes (EWC-Stat rev.4) (EC, 2010), which includes FW with other types of waste, such as garden waste and sludge from washing and the cleaning of food. Member States may, on a voluntary basis, report the three EWC classes containing FW disaggregated to the administrative classification List of Waste (EC, 2010). However, this classification does not capture the amount of FW as a separate category (Eurostat, 2017; Hanssen et al., 2013). A possible way to improve European statistical data on FW would be to update the classification used by Member States for waste reporting, e.g. by reporting FW as a separate category. This would be a long process requiring the involvement of Member States to test the feasibility of data collection.

The monitoring of progress towards FW-related targets, on either generation, prevention, or valorisation, requires the identification of meaningful indicators. Besides the definition of appropriate methods, the definition of an indicator requires the choice of the baseline year on which changes in FW generation or management will be monitored. In the UK, for instance, the analysis of available evidence led WRAP to identify 2007 as baseline year (WRAP, 2014). Other important elements of the indicators are: i) the choice of the unit of measurement, such as mass, calorific value, economic value or greenhouse gas (GHG) emissions; ii) the reporting either in absolute or in percentage terms; iii) the choice of denominator (per capita, total production or total turnover); and iv) the reference to the total mass of FW or only the edible part. Through the ForMat project in Norway (Stensgård and Hanssen, 2016), for example, edible FW has been reported from most of the food chain both with respect to mass, economic value and GHG emissions. This study showed that, whereas the per capita mass of FW was reduced by 12% over six years, the economic value of the FW increased because more expensive food products were bought (Stensgård and Hanssen, 2016). An analysis of FW in six Swedish supermarkets highlighted that fruit and vegetables were by far the most wasted products in terms of mass (86% vs 3% for meat) and economic value (72% for fruit and vegetables vs 12% for meat), whereas the differences were lower when referring to GHG emissions (48% for fruit and vegetables vs 30% for meat). In addition, the monitoring of waste over time highlighted that in some years the total mass of FW increased, whereas the GHG emissions decreased due to lower amounts of wasted meat and other animal-based products (Eriksson, 2015). All of these examples highlight the importance of choosing representative indicators in light of specific policy objectives.

Absolute terms or percentage indicators have both pros and cons. Percentage indicators can be expressed in terms of yearly change or as FW divided by either food purchased, produced or consumed, and are more effectively communicated. However, their interpretation can be confusing, because of variations in the denominators. Percentage indicators do not grasp if the starting point is already a virtuous situation. Setting targets for contexts in which FW is already managed as a priority, indeed, may lead to a trade-off where the required efforts are higher than the benefits in both economic and environmental terms. Considering the European context, the adoption of differentiated percentage targets between Member States, following an approach similar to that adopted for achieving the international GHG emission targets (e.g. Paris Climate Agreement, UNFCCC (2015)), could be an option to take into consideration the extent to which Member States are addressing the FW issue.

On the other hand, absolute indicators calculated in different contexts are difficult to compare and their variation over time may be due to socio-economic and environmental factors, such as reduction of agro-food production or population, rather than to the effectiveness of FW policy design. Comparisons and monitoring over time should preferably be carried out only after normalisation of the results, on the basis of relevant normalisation factors, such as the ones reported by FUSIONS (Møller et al., 2014a, Møller et al., 2014b).

### 3.6 Food waste generation drivers

The analysis of the drivers behind FW generation is important for properly designing the framework for data collection on FW generation, and gives insights on how to structure effective FW reduction strategies.

The generation of FW is influenced by various factors, such as behavioural, technological, product-related, legislative, and societal ones. The relevance of those factors can be highly context-specific and not always predictable (Canali et al., 2016, Secondi et al., 2015). Roodhuyzen et al. (2017), for example, highlighted the fact that different factors can have contradictory effects on FW generation depending on the context in which the study is carried out. This calls for more causal insight analyses to explain contradictions. Therefore, interpretation of the drivers of FW generation is still an open challenge, as is the availability of longitudinal data through which causation analysis could be carried out.

## 4 Conclusions and outlook

The discussion on FW accounting has highlighted several challenges that need to be overcome to ensure robust support for decision-making in relation to FW reduction and valorisation policies and interventions. Some opportunities to overcome these challenges have been identified within existing tools. Building on these opportunities not only can reduce the amount of resources to be invested for FW accounting, but also strengthen the harmonisation of accounting approaches, which was so far lacking in existing accounting studies. Where opportunities were not identified or had a room for improvement, further advancements have been highlighted.

The identified challenges, opportunities and further advancements are strongly interconnected and, although all these elements can contribute to improve FW accounting, the authors of the paper agreed that some of the challenges can be considered of primary importance, such as: i) harmonise FW account guidelines. Existing methodological guidelines have been identified as an opportunity for various challenges for FW accounting. While reducing methodological uncertainties, they contribute to the definition of a robust framework for monitoring the generation of FW, which has a key role in supporting the achievement of the SDG 12.3 FW reduction target; ii) reasonably increase of the quantity and representativeness of primary data. Acknowledging that most of the FSC is characterised by a large number of small entities, increasing the representativeness of the data may impose a considerable economic burden. It is therefore, important to find a balance between the costs of data collection and their representativeness; iii) develop methods for liquid waste accounting. Liquid FW and the accounting of the moisture content of FW along the food chain deserves particular attention. So far, few data are available on liquid FW generation, and considerations on the moisture content of FW are hardly reported in existing studies. However, both elements may considerably influence the accounting process and may affect the monitoring of FW generation over time and in different contexts.

Overall, FW accounting at any geographical scale should be based on a broad understanding of the context in which FW is generated, with a constant focus on the ultimate aim of the accounting exercise.

## References

[bib1] Caldeira, C., Corrado, S., Sala, S., 2017. Food waste accounting. Methodologies, challenges and opportunities. JRC technical report. doi:10.2760/54845. 10.1016/j.gfs.2019.01.002 PMC6472538 31008044

[bib2] Canali M., Amani P., Aramyan L., Gheoldus M., Moates G., Östergren K., Silvennoinen K., Waldron K., Vittuari M. (2016). Food waste drivers in Europe, from identification to possible interventions. Sustainability.

[bib3] Chaboud G., Daviron B. (2017). Food losses and waste: navigating the inconsistencies. Glob. Food Secur..

[bib4] Champions 12. 3, 2017. Guidance on interpreting sustainable development goal target 12.3.

[bib5] Corrado S., Sala S. (2018). Food waste accounting along global and European food supply chains: state of the art and outlook. Waste Manag..

[bib6] Cristobal J., Caldeira C., Corrado S., Sala S. (2018). Techno-economic and profitability analysis of food waste biorefinery concepts at European level. Bioresour. Technol. J..

[bib7] De Laurentiis V., Corrado S., Sala S. (2018). Quantifying household waste of fresh fruit and vegetables in the EU. Waste Manag..

[bib8] Eriksson M. (2015). Prevention and Management of Supermarket Food Waste: With Focus on Reducing Greenhouse Gas Emissions (Doctoral thesis).

[bib9] Eriksson M., Osowski C.P., Malefors C., Björkman J., Eriksson E. (2017). Quantification of food waste in public catering services–a case study from a Swedish municipality. Waste Manag..

[bib10] European Commission (EC), 2010. Commission Regulation (EU) No 849/2010 of 27 September 2010 amending Regulation (EC) No 2150/2002 of the European Parliament and of the Council on waste statistics.

[bib11] European Commission (EC), 2015. Communication from the Commission to the European Parliament, the council, the European economic and social committee and the committee of the regions. Com/2015/0614 final. Closing the loop - an EU action plan for the circular economy.

[bib12] European Commission (EC), 2018. SWD/2018/17 final. Measuring progress towards circular economy in the European Union – Key indicators for a monitoring framework.

[bib13] European Parliament and Council, 2018. Directive (EU) 2018/851 of the European Parliament and of the Council of 30 May 2018 amending Directive 2008/98/EC on waste.

[bib14] European Parliament (EP), 2002. Regulation (EC) No 178/2002 of the European Parliament and of the Council of 28 January 2002 laying down the general principles and requirements of food law, establishing the European Food Safety Authority and laying down procedures in matters of food safety.

[bib15] Eurostat, 2017a. Food Waste Statistics. 〈www.ec.europa.eu/food/sites/food/files/safety/docs/fw_eu-platform_20170925_sub-fwm_pres-03.pdf〉. (Accessed 2 July 2017).

[bib16] Food and Agriculture Organization of the United Nations (FAO), 2011. Global food losses and food waste – extent, causes and prevention.

[bib17] Food and Agriculture Organization of the United Nations (FAO), 2013. Food wastage footprint: impacts on natural resources: summary report.

[bib18] Food Loss and Waste Protocol (FLW Protocol), 2018. Tools and Resources. 〈http://flwprotocol.org/flw-standard/tools-resources/〉. (Accessed 21 February 2018).

[bib19] Food Loss and Waste Protocol (FLW Protocol), 2016. Food Loss and Waste Accounting and Reporting Standard (FLW Standard) – Version 1.0. 〈https://www.wri.org/sites/default/files/REP_FLW_Standard.pdf〉. (Accessed 5 December 2017).

[bib20] Hanssen, O.J., Stenmarck, Å., Dekhtyar, P., O’Connor, C., Östergren, K., 2013. Review of EUROSTATs reporting method and statistics. FUSIONS report. ISBN: 82-7520-706-1 978-82-7520-706-5.

[bib21] Hartikainen H., Mogensen L., Svanes E., Franke U. (2017). Food waste quantification in primary production–the Nordic countries as a case study. Waste Manag..

[bib22] Hendriks C., Obernosterer R., Müller D., Kytzia S., Baccini P., Brunner P.H. (2000). Material flow analysis: a tool to support environmental policy decision making. Case-studies on the city of Vienna and the Swiss lowlands. Local Environ..

[bib23] Kemna, R., Holsteijn, F.H. van, Lee, P., Sims, E., 2016. Complementary research on household refrigeration – optimal food storage conditions in refrigeration appliances. VHK in collaboration with Oakdene Hollins for the European Commission, Brussels. 〈www.ecodesign-fridges.eu〉. (Accessed 11 December 2017).

[bib24] Kummu M., de Moel H., Porkka M., Siebert S., Varis O., Ward P.J. (2012). Lost food, wasted resources: global food supply chain losses and their impacts on freshwater, cropland, and fertiliser use. Sci. Total Environ..

[bib25] Merciai S., Schmidt J. (2018). Methodology for the construction of global multi‐regional hybrid supply and use tables for the EXIOBASE v3 database. J. Ind. Ecol..

[bib26] Møller, H., Hanssen, O.J., Svanes, E., Hartikainen, H., Silvennoinen, K., Gustavsson, J., Östergren, K., Schneider, F., Soethoudt, H., Canali, M., Politano, A., Gaiani, S., Redlingshöfer, B., Moates, G., Waldron, K., Stenmarck, Å., 2014a. Standard approach on quantitative techniques to be used to estimate food waste levels, FUSIONS Report.

[bib27] Møller, H., Hanssen, O.J., Gustavsson, J., Östergren, K., Stenmarck, Å., Dekhtyar, P., 2014b. Report on review of (food) waste reporting methodology and practice. FUSIONS Report. ISBN 82-7520-713-4 978-82-7520-713-3.

[bib28] Östergren, K., Gustavsson, J., Bos-Brouwers, H., Timmermans, T., Hanssen, O.J., Møller, H., Anderson, G., O’Connor, C., Soethoudt, H., Quested, T., Easteal, S., Politano, A., Bellettato, C., Canali, M., Falasconi, L., Gaiani, S., Vittuari, M., Schneider, F., Moates, G., Waldron, K., Redlinghöfer, B., 2014. FUSIONS Definitional Framework for Food Waste – full report. FUSIONS Report.

[bib29] Parry, A., 2017. Measuring Household Food Waste – The UK Experience. Meeting of the EU Platform on Food Losses and Food Waste. 〈https://ec.europa.eu/food/sites/food/files/safety/docs/fw_eu-platform_20170331_wrap-measuring.pdf〉. (Accessed 22 November 2017).

[bib30] Porter S.D., Reay D.S., Higgins P., Bomberg E. (2016). A half-century of production-phase greenhouse gas emissions from food loss & waste in the global food supply chain. Sci. Total Environ..

[bib31] Redlingshöfer B., Coudurier B., Georget M. (2017). Quantifying food loss during primary production and processing in France. J. Clean. Prod..

[bib32] Roodhuyzen D.M.A., Luning P.A., Fogliano V., Steenbekkers L.P.A. (2017). Putting together the puzzle of consumer food waste: towards an integral perspective. Trends Food Sci. Techn..

[bib33] Secondi L., Principato L., Laureti T. (2015). Household food waste behaviour in EU-27 countries: a multilevel analysis. Food Policy.

[bib34] Stenmarck, A., Jensen, C., Quested, T., Moates, G., Buksti, M., Cseh, B., Juul, S., Parry, A., Politano, A., Redlingshofer, B., Scherhaufer, S., Silvennoinen, K., Soethoudt H., Zübert, C., Östergren K., 2016. Estimates of European food waste levels. FUSIONS Report. ISBN 978-91-88319-01-2.

[bib35] Stensgård, A., Hanssen, O.J., 2016. Avoidable Food Waste in Norway 2010-15. Final Report from the ForMat project. Ostfold Research - Research Reports, OR.17.16, Fredrikstad.

[bib36] Tostivint, C., Östergren, K., Quested, T., Soethoudt, J.M., Stenmarck, A., Svanes, E., O’Connor, C., 2016. Food waste quantification manual to monitor food waste amounts and progression. FUSIONS Report.

[bib37] United Nations (UN), 2015. Sustainable Development Goals – 17 Goals to transform our World. 〈www.un.org/sustainabledevelopment/sustainable-development-goals/〉. (Accessed 10 September 2017).

[bib38] United Nations Framework Convention on Climate Change (UNFCC), 2015. Adoption of the Paris Agreement (FCCC/CP/2015/L.9/Rev.1, 2015).

[bib39] United Nations Statistics Division (UNSD), 2017. Work Plans for Tier III Indicators. 〈https://unstats.un.org/sdgs/files/meetings/iaeg-sdgs-meeting-05/TierIII_Work_Plans_03_03_2017.pdf〉. (Accessed 21 February 2018).

[bib40] Van Herpen, E., van der Lans, I., Nijenhuis de Vries, M., Holthuysen, N., Kremer, S., Stijnen, D., 2016. Consumption Life Cycle Contributions. Assessment of practical methodologies for in-home waste measurement. REFRESH Report.

[bib41] WRAP, 2014. UK food waste – Historical changes and how amounts might be influenced in the future. Final report.

[bib42] WRAP, 2016. Food surplus and waste in the UK whole sale grocery, 2015. Research note.

[bib43] WRAP, 2018a. Food waste measurement principles and resources guide. 〈http://www.wrap.org.uk/sites/files/wrap/Food%20waste%20measurement%20principles%20and%20resources.pdf〉. (Accessed 21 February 2018).

[bib44] WRAP, 2018b. Measuring and reporting food waste in hospitality and food service. 〈http://www.wrap.org.uk/content/measuring-and-reporting-food-waste-hospitality-and-food-service〉. (Accessed 21 February 2018).

[bib45] World Research Institute (WRI) and Ministry of Economic Affairs of the Netherlands, 2016. Champions 12.3. 〈www.champions123.org〉. (Accessed 10 September 2017).

[bib46] Xue L., Liu G., Parfitt J., Liu X., Van Herpen E., Stenmarck A., O’Connor C., Östergren K., Cheng S. (2017). Missing food, missing data? A critical review of global food losses and food waste data. Environ. Sci. Technol..

[bib47] Yadav B.K., Jindal V.K. (2007). Water uptake and solid loss during cooking of milled rice (Oryza sativa L.) in relation to its physicochemical properties. J. Food Eng..

